# Neuropilin-1 Identifies a New Subpopulation of TGF-β-Induced Foxp3^+^ Regulatory T Cells With Potent Suppressive Function and Enhanced Stability During Inflammation

**DOI:** 10.3389/fimmu.2022.900139

**Published:** 2022-05-04

**Authors:** Weiqian Chen, Weishan Huang, Youqiu Xue, Ye Chen, Wenbin Qian, Jilin Ma, Avery August, Julie Wang, Song Guo Zheng, Jin Lin

**Affiliations:** ^1^ Division of Rheumatology, The First Affiliated Hospital, Zhejiang University School of Medicine, Hangzhou, China; ^2^ Division of Rheumatology, Department of Medicine, Pennsylvania State University Hershey College of Medicine, Hershey, PA, United States; ^3^ Department of Microbiology and Immunology, College of Veterinary Medicine, Cornell University, Ithaca, NY, United States; ^4^ Department of Pathobiological Sciences, School of Veterinary Medicine, Louisiana State University, LA, United States; ^5^ Department of Clinical Immunology, The Third Affiliated Hospital of Sun Yat-sen University, Guangzhou, China; ^6^ Division of Hematology, The Second Affiliated Hospital, Zhejiang University School of Medicine, Hangzhou, China

**Keywords:** regulatory T cells, TGF-β, Foxp3, neuropilin-1, IL-10

## Abstract

CD4^+^Foxp3^+^ regulatory T cells (Tregs) play a crucial role in preventing autoimmunity and inflammation. There are naturally-derived in the thymus (tTreg), generated extrathymically in the periphery (pTreg), and induced *in vitro* culture (iTreg) with different characteristics of suppressiveness, stability, and plasticity. There is an abundance of published data on neuropilin-1 (Nrp-1) as a tTreg marker, but little data exist on iTreg. The fidelity of Nrp-1 as a tTreg marker and its role in iTreg remains to be explored. This study found that Nrp-1 was expressed by a subset of Foxp3^+^CD4^+^T cells in the central and peripheral lymphoid organs in intact mice, as well as in iTreg. Nrp-1^+^iTreg and Nrp-1^-^iTreg were adoptively transferred into a T cell-mediated colitis model to determine their ability to suppress inflammation. Differences in gene expression between Nrp-1^+^ and Nrp-1^-^iTreg were analyzed by RNA sequencing. We demonstrated that the Nrp-1^+^ subset of the iTreg exhibited enhanced suppressive function and stability compared to the Nrp-1^-^ counterpart both *in vivo* and *in vitro*, partly depending on IL-10. We found that Nrp-1 is not an exclusive marker of tTreg, however, it is a biomarker identifying a new subset of iTreg with enhanced suppressive function, implicating a potential for Nrp-1^+^iTreg cell therapy for autoimmune and inflammatory diseases.

## Introduction

Regulatory T cells (Treg) are crucial for maintaining immune homeostasis, limiting the immune response, and establishing immunological tolerance (1). The transcription factor forkhead box P3 (Foxp3) is a specific marker of Treg. CD4^+^CD25^+^Foxp3^+^Tregs are heterogeneous and can be mainly divided into 3 subsets: thymus-derived naturally occurring Treg (tTreg, also called nTreg), peripherally derived Treg (pTreg), and Treg induced *in vitro* with interleukin-2 (IL-2) and transforming growth factor-β (TGF-β) (iTreg) (2, 3). These three subsets have similar phenotypic characteristics and comparable suppressive function against T cell-mediated immune response and diseases. However, they exhibit certain specific differences in mRNA transcripts and protein expression, epigenetic modification, and stability in the inflammatory milieu. Accurately distinguishing them will help to clarify the biological features and contributions of each Treg subset to peripheral tolerance, autoimmunity, and tumor surveillance. The classical Treg surface markers CD25, GITR, CTLA4, and PD-1 are all expressed on these three Treg subsets, making it extremely difficult to distinguish pTreg and iTreg from tTreg using surface markers. However, two reports suggested neuropilin-1 (Nrp-1) as a promising candidate of tTreg specific surface marker (4, 5).

Nrp-1 is a transmembrane glycoprotein previously known to be involved in axon guidance (6) and angiogenesis (7, 8), which was first found on the surface of tTreg in 2004 (9). It was shown that Nrp-1 was expressed by tTreg *in vivo*, but not by pTreg driven by antigenic stimulation or converted under homeostatic conditions in murine models (4, 5). In contrast, the expression of Nrp-1 was found upregulated in TGF-β-induced Treg *in vitro (* 4). It was also noticed that Nrp-1 is not a marker of human nature FOXP3^+^Treg, but can be induced in human blood T cells upon *in vitro* TCR activation (10). Sarris found that Nrp-1 strengthened the contacts between Treg and antigen-presenting cells (11). Similar to Nrp-1, Helios has been reported as a potential marker for tTreg (12–15). iTreg generated from both human and mice CD4^+^T cells could express Helios (16). Helios is also known as a marker of T cell activation and proliferation (17, 18).

Recently, several literatures described the potential use of iTreg as a therapeutic strategy for autoimmune diseases. One study showed that polyclonal iTreg slowed diabetes progression, prolonging the survival of non-obese diabetic mice (19). Collagen type II-specific iTreg was better than nTreg in suppressing arthritis, partly by inhibiting the development of Th17 cells (20). Furthermore, iTreg could suppress the main features of asthma (21). Although iTreg had a suppressive function, its long efficacy was less acceptable. Thus, it is difficult to identify potent a suppressive iTreg based on surface markers (1).

Our study aimed to systematically explore the role of Nrp-1 and Helios in Treg subsets with a focus on the role of Npr-1 in iTreg function and stability. We found that Nrp-1 and Helios are not exclusive markers of the tTreg subset. The expression of Nrp-1 on the surface of iTreg enables the identification and isolation of an iTreg subset that has superior a suppressive function under inflammatory conditions.

## Material And Methods

### Mice

Male C57BL/6 (B6), C57BL/6 thy1.1, B6.129S7-*Rag1^tm1Mom^*/J (Rag1^-/-^), B6 Foxp3-RFP mice and CD4^cre^ B6 mice were purchased from the Jackson Laboratory. B6 Foxp3-GFP knockin mice were generously provided by Dr. Talil Chatilla (University of California Los Angeles). CD4^cre^/Nrp1^flox/flox^ B6 mice were generated by crossing the two parent strains at Cornell University. All mice were kept in the specific pathogen-free (SPF) condition. 7-8 weeks age mice were chosen for the experiment.

### The Generation of CD4^+^ Induced Regulatory T (iTreg), Th1, Th2, and Th17 Cells

Naïve splenic CD4^+^CD62L^+^CD25^-^CD44^low^T cells were acquired by negative selection *via* the auto-MACS method (22, 23). Briefly, enriched T cells were first stained with biotin-conjugated anti-CD8a, -CD25, -CD11b, -CD49b, -CD11c, and -B220 mAbs and then washed and combined with anti-biotin microbeads (Miltenyi Biotec, Auburn, CA, USA). After they passed through the MACS separation columns, the negative exports were collected as CD4^+^CD25^-^cells. Subsequently, naive CD4^+^CD25^-^CD62L^+^CD44^low^ T cells were positively selected from the enriched CD4^+^CD25^-^T-cell fraction by the anti-CD62L microbeads. For iTreg differentiation, 0.2x10^6^ cells were cultured in 96-well plates and stimulated with anti-CD3/CD28 microbeads (1 bead per 5 cells, Invitrogen) in the presence of IL-2 (10ng/ml, R&D) with (CD4^+^iTreg) or without (CD4_med_) TGF-β (2ng/ml) for 3 days. Other 0.2x10^6^ naïve CD4^+^T cells were cultured with irradiated APCs (1:1 ratio of APCs to Naïve CD4^+^ T cells) in the presence of 1 μg/ml soluble anti-CD3 and 1 μg/ml anti-CD28, together with different antibodies and cytokines in 96-well plates. For Th1 cell differentiation, 10 ng/ml recombinant murine IL-12 (rm-IL-12, eBioscience) and 10 μg/ml anti-IL-4 (Biolegend) were used. For Th2 cell differentiation, 10 ng/ml rm-IL-4 (R&D Systems), and 10 μg/ml anti-interferon-γ (anti-IFN-γ, Biolegend) were used. For Th17 cell differentiation, 30 ng/ml IL-6 (R&D Systems), 1 ng/ml TGF-β (R&D Systems), 10 μg/ml anti-IFN-γ (Biolegend), and 10 μg/ml anti-IL-4 (Biolegend) were added. RPMI 1640 medium supplemented with 100 U/ml penicillin, 100 mg/ml streptomycin, and 10mM HEPES (Invitrogen) and 10% heat-inactivated FCS (HyClone Laboratories) was used for all cultures. Cells were harvested and stained with different antibodies.

### Flow Cytometry

The following fluorescence conjugated mouse antibodies were used for flow cytometric analysis: From Biolegend: anti-CD4 (GK1.5), CD25 (3C7), CD8 (53-6.7), B220 (RA3-6B2), CD44 (IM7), CD11c (N418), CD11b (M1/70), NK1.1 (PK136), Helios (22F6), IFN-γ (XMG1.2), IL-17a (TC11-18H10.1), IL-4 (11B11), IL-10 (JES5-16E3) IL-10R (1B1.3a); From R&D Systems: Neuropilin−1 (FAB5994A). Cell subset was stained with mAbs and isotype control and analyzed by a FACS Calibur flow cytometer. For intracellular staining, such as IFN-γ and IL-17a, cells were first stained with surface marker CD4, and further fixed, and permeabilized for intracellular staining.

### *In Vitro* Suppression Assays

Freshly isolated 0.2x10^6^ T cells (responder cells) labeled with CFSE were stimulated with anti-CD3 mAb (0.025 μg/mL) and irradiated APCs (30 Gy, 1:1 ratio) for 3 days, with or without iTreg generated as described above. The ratio of Treg/T cells was 1:2-1:16. T-cell proliferation was determined by the CFSE dilution rate after 3 days of culture.

### Foxp3(GFP)^+^Nrp-1^+^CD4^+^T Cells Conversion *In Vivo*

When the 90.1^+^CD4^+^iTreg were cultured for 3 days and harvested, they were sorted into 90.1^+^Foxp3-GFP^+^Nrp-1^+^CD4^+^T or 90.1^+^Foxp3-GFP^+^Nrp-1^-^CD4^+^T cells. Anti-CD3/CD28 microbeads were removed. For *in vivo* conversion, 0.5x10^6^ 90.1^+^Foxp3-GFP^+^Nrp-1^+^CD4^+^T or 90.1^+^Foxp3-GFP^+^Nrp-1^-^CD4^+^T cells were adoptively transferred into Rag1^-/-^ mice. 4, 7, and 12 days later, 90.1^+^CD4^+^T cells from the spleen were stained for Foxp3-GFP, IFN-γ, and IL-17a.

### T Cell-Induced Colitis

Naïve CD4^+^CD45RB^hi^ T cells were purified (>98%) from spleens of C57BL/6 Foxp3^gfP^ mice *via* FACS sorting (LSR II, BD). Naïve CD4^+^CD45RB^hi^ T cell suspensions were washed in sterile PBS, and age- and sex-matched C57BL/6 Rag1^-/-^ recipient mice received 4x10^5^ CD4^+^CD45RB^hi^ T cells by i.p. injection.

For co-transfer experiments, 1:2 mixtures of CD90.1^+^Foxp3-GFP^+^Nrp-1^+^CD4^+^T or CD90.1^+^Foxp3-GFP^-^Nrp-1^+^CD4^+^T or CD90.1^+^Foxp3-GFP^-^Nrp-1^-^CD4^+^T or CD90.1^+^Foxp3 -GFP^+^Nrp-1^-^CD4^+^T and CD90.2^+^CD4^+^CD45RB^hi^ T cells were injected i.p. (total cell number=4x10^5^) into C57BL/6 Rag1^-/-^. Mice were sacrificed when symptoms of clinical disease (weight loss and/or diarrhea) developed in control groups, 6-8 weeks after cells transfer unless otherwise indicated. Samples of the cecum and proximal, mid, and distal colon were prepared as previously described, and inflammation was graded according to a scoring system (23, 24).

### RNA Sequence Analysis

Naïve CD4^+^T cells were isolated from the spleen of B6 Foxp3-RFP mice. Nrp-1^+^Foxp3-^+^iTreg and Nrp-1^-^Foxp3-^+^iTreg cells were sorted from iTreg cells on day 3 based on Nrp-1 and Foxp3-RFP expression. Thymus-derived nTreg cells from B6 Foxp3-RFP mice were set as a control. Total RNA was prepared from the above three Treg using the RNeasy Mini Plus Kit (Qiagen). Directional RNA-seq libraries were prepared using the NEBNext Ultra Directional RNA Library Prep Kit for Illumina (New England Biolabs), with initial polyA+ isolation, by the RNA Sequencing Core at Cornell University. Sequencing was performed on Illumina HiSeq 1500, and raw data were processed on the CLC Genomics Workbench v 11.0.1. mRNA profiles were calculated with Cufflinks software and expressed as FPKM (fragments per kilobase of exon model per million mapped fragments). Genes that are significantly altered (|FC|≥1, *P*<0.05) in Nrp-1^+^ iTreg vs Nrp-1^-^ iTreg cells, are used for Gene Set Enrichment Analysis (GSEA).

### Bisulfite Sequencing

We harvested genomic DNA from Nrp-1^+^Foxp3-GFP^+^iTreg and Nrp-1^-^Foxp3-GFP^+^iTreg cells using the DNeasy blood & tissue extraction kit (Qiagen) and conducted a bisulfite conversion using an EZ DNA Methylation kit (Zymo Research) following the manufacturer’s protocol. Purified bisulfite-treated DNA was amplified by PCR using a pair of primers to mouse Foxp3 TSDR: 5′- AGAGGTTGAAGGAGGAGTATTT -3′ and 5′- ACTATCTATCCAATTCCCCAAC -3′. The PCR products were purified using ExoSAP-IT PCR Product Cleanup Kit and were sequenced by GeneWIZ company. All sequencing results of the bisulfite converted TSDR region were analyzed on the Bisulfite sequencing data presentation and compilation (BDPC) DNA methylation analysis platform.

### Statistics

Data are expressed as Mean ± SEM unless otherwise indicated. Data were analyzed using the unpaired t-tests (Mann-Whitney) or paired t-tests for comparison between two groups or ANOVA for comparison among multiple groups as appropriate. Differences were considered statistically significant when *p*<0.05.

## Results

### Both Nrp-1 and Helios Were Highly Expressed in iTreg Subset

Current studies debate the value of Nrp-1 and Helios specificity on tTreg. Using Foxp3^gfp^ reporter mice, we systematically investigated their expression profile. Nrp-1 was expressed on CD4^+^T, DC, and NK cells, which was consistent with similar findings in the reference (25), while Helios was mostly expressed on CD4^+^T cells (**Supplemental Figure 1**). Among CD4^+^T cells in the thymus, Nrp-1 was exclusively expressed in CD4^+^Foxp3^+^T cells albeit its level was a little lower than that of Helios. However, Helios was also expressed on CD4^+^Foxp3^-^T cells (**Supplemental Figure 2**), thus, Nrp-1 would be better to identify tTreg than Helios.

We also studied the expression of Nrp-1 and Helios in the peripheral lymph tissues. As shown in **Supplemental Figures 2A, C**, similar levels of Nrp-1 were observed in CD4^+^Foxp3^+^T cells isolated from the lymph node, spleen, and circulating blood compared to the thymus. Helios was also substantially expressed on peripheral CD4^+^Foxp3^+^ cells. Given that peripheral CD4^+^Foxp3^+^ Treg could be mixed with tTreg and pTreg, Neither Nrp-1 nor Helios may distinguish nTreg from pTreg cells.

**Figure 1:**
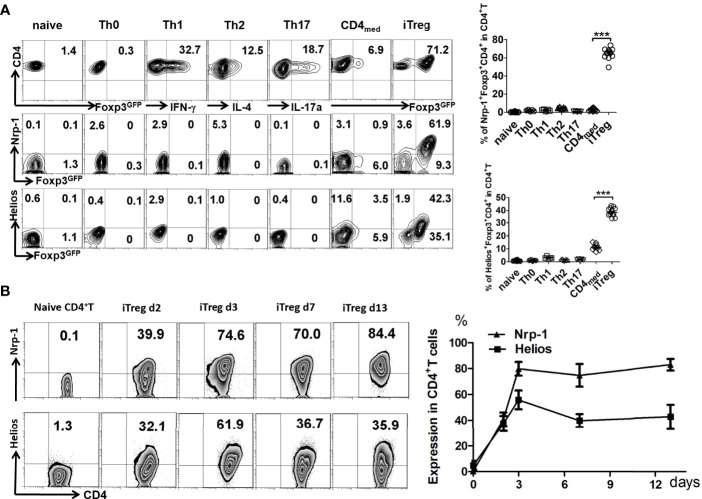
Nrp-1 was highly expressed in induced CD4^+^ Treg *in vitro*. **(A)** The expression of Nrp-1 and Helios were measured in naïve CD4^+^T cells, Th0, Th1, Th2, Th17, CD4_med_, or iTreg by flow cytometry. Nrp-1, Helios, and Foxp3-GFP were analyzed together. Representative FACS plots and the summarized data (right) of three separated experiments were shown. (***p<0.001). **(B)** Dynamic Nrp-1 and Helios expression in iTreg cells *in vitro at different days.* Representative data was presented.

We found that Nrp-1 and Helios were both negative on naïve CD4^+^T cells. After T cell receptor (TCR) stimulation, 12% of CD4^+^T cells expressed Helios, but Nrp-1 was lower-expressed on activated CD4^+^T cells. After treatment with TGF-β, >75% of CD4^+^T cells expressed Foxp3 that was considered as iTreg. 55% of iTreg expressed Helios and 87% were both Nrp-1^+^Foxp3^+^CD4^+^T (**Figure 1A**). Both Nrp-1 and Helios were hardly expressed on Th1, Th2, and Th17 cells differentiated *in vitro*. Nrp-1 expression increased after 2 days of culture *in vitro*, and was still higher-expressed in iTreg *in vitro* after 13 days (**Figure 1B**).

### Nrp-1^+^ iTreg Was More Stable Than Nrp-1^-^ iTreg *In Vitro* and *In Vivo*

**Figure 2:**
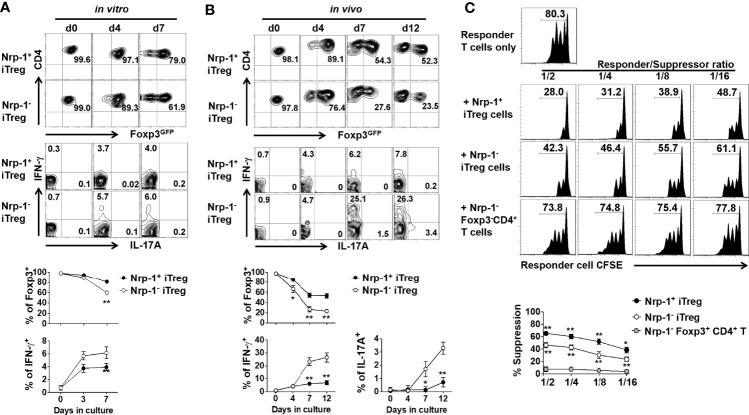
Foxp3-GFP^+^Nrp-1^+^iTreg was more stable *in vitro* and *in vivo with* a prior suppression *in vitro*. **(A)** Foxp3-GFP^+^Nrp-1^+^iTreg and Foxp3-GFP^+^Nrp-1^-^iTreg were sorted from iTreg, and then cultured with CD3CD28 beads (cells/beads 1/5)+rhIL-2 (10ng/ml) for several days *in vitro*. Cells were harvested at indicated days; Foxp3-GFP, IFN-γ, and IL-17a were measured on GFP^+^Nrp-1^+^iTreg and GFP^+^Nrp-1^-^iTreg by FACS. Representative FACS plots and the summarized data of three separated experiments were shown. **(B)** GFP^+^Nrp-1^+^iTreg and GFP^+^Nrp-1^-^iTreg were transferred into Rag1^-/-^ mice. Mice were sacrificed, spleen cells were harvested at indicated days; Foxp3-GFP, IFN-γ, and IL-17a were measured on GFP^+^Nrp-1^+^iTreg and GFP^+^Nrp-1^-^iTreg by FACS. Representative FACS plots and the summarized data of three separated experiments were shown. The data indicated Mean ± SEM of two separate experiments (n=6) (*p<0.05, **p<0.01). **(C)** Freshly isolated T cells (responder cells) labeled with CFSE were stimulated with anti-CD3 mAb (0.025 μg/mL) and irradiated APCs (30 Gy, 1:1 ratio) for 3 days, with or without Foxp3GFP^+^Nrp-1^+^iTreg or Foxp3GFP^+^Nrp-1^-^iTreg or Foxp3GFP^-^Nrp-1^-^CD4^+^T cells. (The ratio of Treg: T cells was 1:2-1:16) T-cell proliferation was determined by the CFSE dilution rate after 3 days of culture. The data indicate the Mean ± SEM of 3 separated experiments (*p<0.05, **p<0.01).

Given that Nrp-1 was highly expressed on iTreg but not activated CD4^+^ cells, we chose to determine the biological significance of Nrp-1 expression on iTreg. Foxp3 stability is closely associated with Treg functionality, we therefore compared the stability of two cell populations *in vitro* and *in vivo*. Firstly, iTreg was induced and then the Nrp-1^+^ and Nrp-1^-^Foxp3^+^ subsets were sorted and re-stimulated with TCR and IL-2 *in vitro*. The Foxp3 level of Nrp-1^+^iTreg was almost maintained from day 4 to day 7, while Nrp-1^-^iTreg significantly lost Foxp3 on day 7. Neither population produced IL-17A, however, Nrp-1^-^ but not Nrp-1^+^iTreg began to produce IFN-γ (**Figure 2A**).

We further evaluated the stability of these two subsets *in vivo*. Both the Nrp-1^+^ and Nrp-1^-^ iTreg subsets were adoptively transferred into Rag1^-/-^ mice, and we observed that Nrp-1^+^ iTreg had a high Foxp3 expression on day 4, which was maintained at 52.3% on day 12 after cell transfer. Conversely, Nrp-1^-^ iTreg significantly reduced Foxp3 on day 4 and dramatically lost Foxp3 expression between days 7-12 after cell transfer. Few Nrp-1^+^iTreg produced IFN-γ but not IL-17A; conversely, 25-30% of Nrp-1^-^ iTreg produced IFN-γ, and 3-4% produced IL-17A (**Figure 2B**).

### Nrp-1^+^ iTreg Displayed Superior Functional Activity *In Vitro* and *In Vivo*

In order to determine the functional significance of Nrp-1 expression on iTreg, we compared the suppressive activity of both iTreg subpopulations. Using a standard *in vitro* suppression system as previously described (26), the suppression exerted by the Nrp-1^+^iTreg against T cell proliferation was superior to the Nrp-1^-^ iTreg subset at the ratios (Treg to T effector cells) of 1:2 to 1:16 (**Figure 2C**).

**Figure 3:**
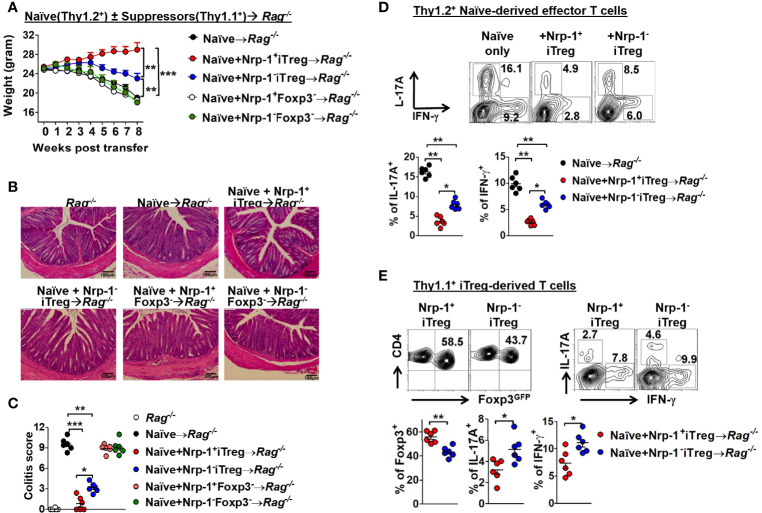
Foxp3-GFP^+^Nrp-1^+^iTreg had prior suppression than Foxp3-GFP^+^Nrp-1^-^iTreg *in vivo.* C57BL/6 Rag1^-/-^ mice were transferred with 4x10^5^ CD4^+^CD25^-^CD45RB^hi^ T cells from C57BL/6 WT mice. Thy1.1 GFP^+^Nrp-1^+^iTreg or GFP^+^Nrp-1^-^iTreg or GFP^-^Nrp-1^+^CD4^+^T or GFP^-^Nrp-1^-^CD4^+^T cells were co-transferred with congenetic CD4^+^CD25^-^CD45RB^hi^ T cells into rag1^-/-^ mice. **(A)** Representative weight loss curve, shown as a percentage of initial weight. **(B, C)** Intestinal inflammation scores for the colon. **(D)** IFN-γ and IL-17a were detected in CD4^+^T cell derived from Thy1.2^+^ naïve T cells in the mesenteric lymph node (MLN). **(E)** Foxp3-GFP, IFN-γ, and IL-17a were detected in GFP^+^Nrp-1^+^iTreg or GFP^+^Nrp-1^-^iTreg from MLN; data were summarized (below). The data indicate the Mean ± SEM of 3 separated experiments (n=6 mice/group) (*p<0.05, **p<0.01, ***p<0.001).

We further developed an *in vivo* colitis model (23) to validate this result. Four CD4^+^T cell populations including Nrp-1^+^ and Nrp-1^-^ iTreg; Nrp-1^+^GFP^-^ and Nrp-1^-^GFP^-^ cells were co-transferred with naïve CD4^+^CD45RB^hi^ T cells to Rag1^-/-^ mice. As expected, two GFP^-^ cell populations failed to suppress colitis, but both iTreg subpopulations displayed suppression. However, Nrp-1^+^iTreg almost completely suppressed the onset and progression of colitis including weight loss and intestine inflammation pathology with a significantly better effect than Nrp-1^-^iTreg (**Figures 3A** **–C**). Accordingly, neither GFP^-^ cells suppressed Th1 and Th17 development, while both iTreg subsets significantly suppressed the development of two pathogenic cells (**Figure 3D**). Consistently, Nrp-1^+^iTreg had a more potent ability to suppress Th1/Th17 cells in colitis (**Figure 3D**). Eight weeks after transfer with naïve CD4^+^CD45RB^hi^ T cells into Rag1^-/-^ mice, Nrp-1^+^iTreg itself maintained relatively higher Foxp3 expression than Nrp-1^-^iTreg. Furthermore, Nrp-1^+^iTreg had lower expression of IFN-γ and IL-17a (**Figure 3E**). It suggested that Nrp-1^+^iTreg suppressed the T cell-mediated intestinal inflammation with stronger stability in the inflammatory milieu *in vivo*.

### Nrp1^+^ iTreg and Nrp1^-^ iTreg Were Distinct Types by RNA Sequence Analysis

**Figure 4:**
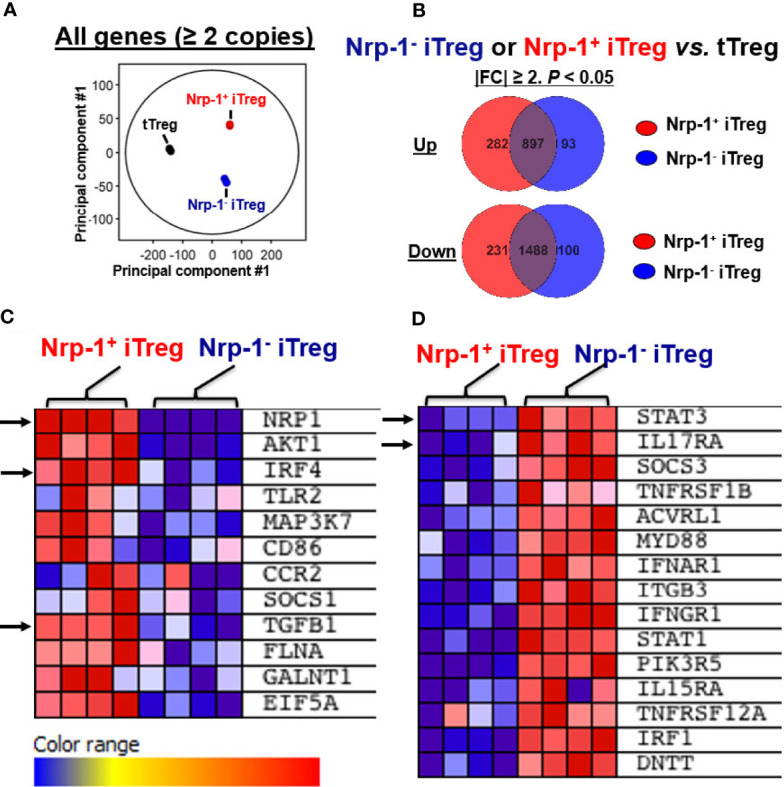
Nrp1^+^iTreg and Nrp1^-^iTreg are distinct by RNA sequence analysis. **(A)** Principal component analysis revealed that nTreg (3 replicates), Nrp1^+^iTreg (4 replicates), and Nrp1^-^iTreg (4 replicates) are distinct. **(B)** Different gene numbers are demonstrated with more than 2 fold changes (FC) in Nrp1^+^iTreg or Nrp1^-^iTreg *vs.* nTreg. Nrp1^+^iTreg and Nrp1^-^iTreg gene numbers are shared or distinct by Mann-Whitney unpaired analysis (*p*<0.05). **(C, D)** some significantly altered genes (|FC|≥1, *p*<0.05) in Nrp1^+^iTreg vs Nrp1^-^iTreg were shown by Gene Set Enrichment Analysis (GSEA).

Next, we performed an RNA sequence analysis. The principal component analysis revealed that nTreg, Nrp1^+^ iTreg, and Nrp1^-^ iTreg were distinct types (**Figure 4A**). Different gene numbers are detected with more than 2 FC in Nrp1^+^ iTreg or Nrp1^-^ iTreg *vs.* nTreg. Nrp1^+^ iTreg and Nrp1^-^ iTreg gene numbers are shared or distinct (**Figure 4B**). We identified the top 50 significantly altered genes (FC≥2) in Nrp1^+^ iTreg vs Nrp1^-^ iTreg. Interestingly, interferon regulatory factor 4 (IRF4) and TGFB1 were increased in the Nrp-1^+^iTreg, while the signal transducer and activator of transcription 3 (STAT3), and interleukin 17 Receptor A (IL17RA) were highly expressed in Nrp-1^-^iTreg (**Figures 4C, D**).

### IL-10 May Account for the Superior Functional Activity of Nrp-1^+^ iTreg *In Vitro*

**Figure 5:**
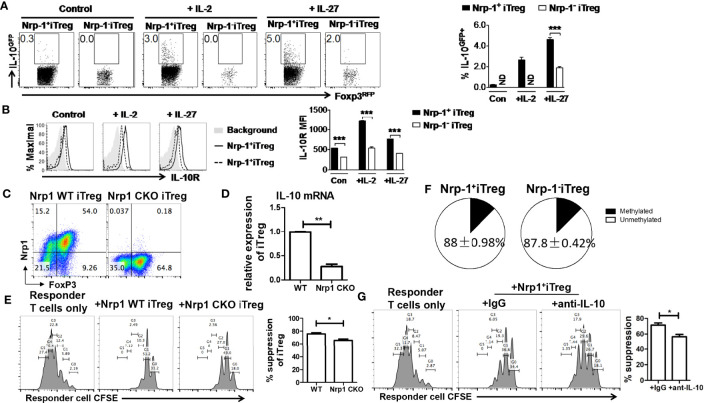
IL-10 may be account for a superior functional activity of Nrp-1^+^ iTreg *in vitro*. **(A, B)** After resting for 4 days, Nrp-1^+^iTreg and Nrp-1^-^iTreg were restimulated with mitomycin-C treated APC (Rag-/-) in the presence of 1 ug/ml anti-CD3/28. Cells were analyzed ~50 hours post restimulation. Control: no supplemental cytokines; +IL-2: + 40U/ml rh-IL-2; +IL-27: + 20ng/ml rm-IL-27. Cells were analyzed ~ 50 hours post restimulation. IL-10 and IL-10R were measured in Nrp-1^+^iTreg and Nrp-1^-^iTreg. **(C–E)** CD4^cre^/Nrp1^flox/flox^ mice were generated to conditional knockout Nrp-1 (Nrp-1 CKO) in CD4^+^T cells. CD4^cre^ mice were set as wild-type (WT) control. Nrp-1 CKO iTreg and WT iTreg were generated from naïve CD4^+^T cells in Nrp-1 CKO and Nrp-1 WT mice respectively. Foxp3 expression was equivalent in Nrp-1 CKO iTreg and WT iTreg. IL-10 mRNA expression was much higher in WT iTreg than that in Nrp-1 CKO iTreg (n=5). There was an inferior functional activity of Nrp-1 CKO iTreg in suppressing CD8^+^T cells (Ratio 1/1) *in vitro* (n=5) by an analysis of flow-based suppression assay. Representative plots and the summarized data of different experiments were shown. The data indicate the Mean ± SEM of 3 separated experiments (*p<0.05, **p<0.01, ***p<0.001). **(F)** The methylation levels were detected by primers designed for the TSDR region in Nrp-1^+^iTreg (n=4) and Nrp-1^-^iTreg (n=4). Nrp-1^+^iTreg and Nrp-1^-^iTreg had similar DNA methylation. **(G)** Anti-IL-10 antibody or IgG were added in the suppression system of Nrp-1^+^iTreg against CD8^+^T cells (Ratio 1/1) *in vitro* (n=4) by an analysis of flow-based suppression assay (*p<0.05).

The inhibitory effect of Treg is partly dependent on IL-10. We also tested IL-10 and IL-10R expression in iTreg. In the resting state, Nrp-1^+^iTreg and Nrp-1^-^iTreg lowly expressed IL-10 (not shown). After resting for 4 days and reculturing with IL-2 or IL-27 that can promote the IL-10 expression in CD4^+^T cells (27), IL-10 and IL-10R were higher in Nrp-1^+^iTreg than in Nrp-1^-^iTreg (**Figures 5A, B**). IL-10R expression was also much higher in resting Nrp-1^+^iTreg than in Nrp-1^-^iTreg (**Figure 5B**).

We further generated CD4^cre^/Nrp1^flox/flox^ mice to conditional knockout Nrp-1 (Nrp-1 CKO) in CD4^+^T cells. CD4^cre^ mice were set as wild-type (WT) control. We found that Foxp3 expression was equivalent in Nrp-1 CKO iTreg and WT iTreg (**Figure 5C**). However, IL-10 mRNA expression was much higher in WT iTreg than that in Nrp-1 CKO iTreg (**Figure 5D**), Nrp-1 CKO iTreg had an inferior functional activity *in vitro* (**Figure 5E**). Furthermore, blocking experiments demonstrated that anti-IL-10 antibody impaired the suppression of Nrp-1^+^iTreg against the proliferation of CD8^+^T cells *in vitro* (**Figure 5G**).

## Discussion

Two important works found that Nrp-1 is expressed at high levels on most nTreg; in contrast, its expression levels in mucosa-generated iTreg and other noninflammatory iTreg were lower (4, 5). Abundant Nrp-1-expressing Treg was found within tumors and inflamed tissues (4). We showed that Nrp-1 was higher-expressed in CD4^+^Foxp3^+^T cells from the lymph node, spleen, and blood, while relative lower-expressed in CD4^+^Foxp3^+^T cells from the thymus. This will raise a question of why Nrp-1 is selectively higher-expressed in peripheral lymphoid organs, but not really in thymus nTreg. A recent research found that Nrp-1 could not identify the nTreg of intrathymic origin by analyzing the T cell repertoire (28). Nrp-1 was abundant among Tregs in peripheral and mesenteric lymph nodes as well as the colon. Some induced Tregs mixed in peripheral CD4^+^Foxp3^+^T cells may be one answer to this question: Is Nrp-1 a marker for induced Treg?

We demonstrated that Nrp-1 expression increased after 2 days of culture *in vitro*, and was still higher-expressed in TGF-β-iTreg (cultured in the presence of TGF-β, TCR, and IL-2) *in vitro* days 13; however, Nrp-1 was lower-expressed in CD4_med_T cells (presence of TCR and IL-2). Weiss, et al. confirmed that Nrp-1 was upregulated in TGF-β-induced Treg *in vitro*, but this was thought of as a transient phenomenon. They induced iTreg cultured with plate-bound anti-CD3 and anti-CD28, IL-2, and TGF-β, and cells were transferred to new wells on day 3. Nrp-1 expression was reduced after the withdrawal of TGF-β (4). They demonstrated the TGF-β mediated control on Nrp-1 expression *via* differentiation of T helper cells using different TGF-β conditions as well as by using TGF-β receptor II conditional knockout mice (4). We further found that the TGF-β signal inhibitor (ALK5i) shut down the Nrp-1 expression in iTreg *in vitro* (**Supplemental Figure 3**). Another group reported that Nrp-1 was lower expressed on iTreg *in vivo* and *in vitro (* 5); they generated iTreg using CD4^+^CD25^-^Foxp3^-^T cells stimulated *in vitro* with anti-CD3 plus anti-CD28 or with irradiated spleen cells in the presence of TGF-β. Meanwhile, IL-2 was not added in their protocol (5). We induced iTreg using IL-2, TGF-β, and anti-CD3/CD28 beads, which can supply instant TCR signals to allow iTreg to survive. IL-2 signaling was important for Nrp-1 expression in Treg (29). It suggested TGF-β can promote the expression of Nrp-1 in iTreg based on TCR and IL-2 signaling. Recent studies demonstrated that TGF-β can also promote the expression of Nrp-1 in lung type II innate lymphoid cells (ILC2) (30). TGF-β can induce the expression of transcription factor SP1 (31), which can upregulate Nrp-1 expression by binding to the Nrp-1 promoter (32). We therefore speculate that Nrp-1 expression in iTreg may be under the control of TGF-β through SP1.

Herein, we demonstrated that Nrp-1^+^iTreg is more stable *in vivo* and *in vitro*, even in an inflammatory state, resistant to conversion to Th1 and Th17 cells, gaining a strong suppressive activity. The DNA demethylation of conserved element within the Foxp3 locus named TSDR (Treg-specific demethylated region) was related to the stability of Treg cells. This region of nTreg is in a non-methylated state, but highly methylated in iTreg (33). The methylation levels were detected by primers designed for the TSDR region, and we found that Nrp-1^+^iTreg and Nrp-1^-^iTreg had a similar DNA methylation (**Figure 5F**), suggesting that the methylation of Foxp3 did not determine the stability of Nrp-1^+^iTreg. The semaphorin-4a(Sema4a)/Nrp-1 interaction recruited phosphatase and tensin homolog (PTEN) and suppressed Akt, consequently maintaining nTreg stability (34). However, this was unknown in iTreg.

Previous studies have confirmed that TGF-β promotes downstream SMAD2/3 phosphorylation and Foxp3 expression through its receptors (Tβ RI and Tβ RII), thus maintaining iTreg stability. Nrp-1 was similar to TGF-β receptors Tβ RI and Tβ RII. Nrp-1 has an affinity for two receptors, acting as a co-receptor for TGF-β to enhance the TGF-β signaling (35). Chuckran, et al. found that Nrp-1 can promote hepatocellular inflammation and fibrosis *via* the TGF-β-mediated SMAD signaling pathway (25). We found that TGF-β1 mRNA expression was increased in Nrp-1^+^iTreg by RNAseq analysis. This may create a positive loop, in which Nrp-1^+^iTreg displays higher TGF-β signaling, further supporting the Nrp-1 function. Nrp-1 may further promote the stability of iTreg *via* the TGF-β-mediated SMAD signaling. On the other hand, Nrp-1^-^iTreg lacks the positive loop mechanism and responds to TGF-β without the enhancement. Mechanistically, the Nrp-1 may serve as a coordinator, which is also important for TGF-β-induced iTreg.

It was reported that iTreg is resistant to Th17 conversion by IL-6 under inflammatory state, because IL-2 and TGF-β downregulate IL-6 receptor expression and IL-6 signaling (26). Our RNAseq analysis demonstrated that IL17RA and STAT3 (downstream of IL-6 signaling), which was related to Th17 cell differentiation, were lower-expressed in Nrp-1^+^iTreg. This suggested that Nrp-1^+^iTreg may exhibit non-plasticity in an inflammatory milieu.

Furthermore, we found that Nrp-1^+^iTreg displayed superior suppression against T cell proliferation *in vitro*. Nrp-1^+^iTreg could completely prevent colitis development, while Nrp-1^-^iTreg exerted only half control of colitis disease. Thus, Nrp-1^+^iTreg is a crucial subset with high suppressive activity and stability. We further found that IL-10 and IL-10R were expressed higher in Nrp-1^+^iTreg than in Nrp-1^-^iTreg. We considered that Nrp-1^+^iTreg had a suppressive function partly depending on IL-10 signaling.

Schmitt EG confirmed that iTreg controlled inflammation by producing IL-10 (36, 37). IL-10 deficiency impairs Nrp-1^+^nTreg function, promotes Th1 and Th17 response (38). One group found that the CD4^+^Nrp-1^+^T cells express greater amounts of IL-10 and show suppressive function. Sema3A, the binding receptor of Nrp-1, acted directly on CD4^+^Nrp-1^+^T cells, promoted IL-10 production, and affected their function (39). Using Nrp-1 CKO mice, we found that IL-10 mRNA expression was much higher in WT iTreg than that in Nrp-1 CKO iTreg. There was an inferior functional activity of Nrp-1 CKO iTreg *in vitro*, and the suppression of Nrp-1^+^iTreg against the proliferation of CD8^+^T cells was dependent on IL-10 *in vitro.* These results suggested that IL-10 signaling contributed to a superior functional activity of Nrp-1^+^ iTreg. Another group found that Nrp-1 KO nTreg had a deficient suppressive function and was defective in IL-10 production (40). We further found that IRF4 was highly expressed in Nrp-1^+^iTreg by RNAseq analysis. The transcription factor IRF4, cooperating with Foxp3, plays an important role in natural Treg differentiation and function (41, 42). IRF4 regulated IL-10 expression in Treg through the remodeling of chromatin at the *IL10* locus (41). IRF4 and Nrp-1 were both functionally involved in CD8^+^T cells (25). We assume that IRF4 will enable Nrp-1^+^iTreg with a higher amount of IL-10 and stronger regulatory function.

Importantly, Nrp-1 is required for Treg to limit anti-tumor immune responses and to cure established inflammatory colitis (34, 43, 44), but is dispensable for the suppression of autoimmunity and maintenance of immune homeostasis (34). The number of nTreg is relatively small in peripheral blood, and it takes a long time to expand *in vitro* before infusion of these cells into recipients. As polyclonal cells, iTreg can be massively expanded from naïve CD4^+^T cells *in vitro*, and it is easy to obtain the number of cells in therapeutic doses, suggesting that iTreg based therapy is a good choice for the treatment of autoimmune diseases.

There are some limitations of this study. We do not complete a protein staining of TGF-β, IRF4, IL17RA, and STAT3. How does IRF4 regulate the IL-10 expression and the function of Nrp-1^+^iTreg are unknown. The role of Nrp-1 in the function of human iTreg has not been mentioned.

In all, our data confirmed that Nrp-1 is a good marker to identify functional iTreg, as Nrp-1^+^iTreg showed a greater suppressive activity and powerful function to maintain the immune homeostasis. These findings may provide a novel strategy for treating autoimmune diseases.

## Data Availability Statement

The data supporting the findings of this study are available from the corresponding author upon reasonable request. The RNA seq data presented in the study are deposited in the GEO repository (https://www.ncbi.nlm.nih.gov/geo/), accession number GSE201416.

## Ethics Statement

The animal study was reviewed and approved by Penn State University and Cornell University for the Use and Care of Animals. Written informed consent was obtained from the owners for the participation of their animals in this study.

## Author Contributions

SZ and JL conceived the study. WC, WH, YX, YC, WQ, JM, AA, and JW performed the experiments. WC, WH, SZ, and JL analyzed the data. WC, WH, and SZ wrote the manuscript. All authors contributed to the article and approved the submitted version.

## Funding

This work was supported in part by grants from Natural Science Foundation of China (82171768, 82001727), the fellowship of China Postdoctoral Science Foundation (2021TQ0377).

## Conflict of Interest

The authors declare that the research was conducted in the absence of any commercial or financial relationships that could be construed as a potential conflict of interest.

## Publisher’s Note

All claims expressed in this article are solely those of the authors and do not necessarily represent those of their affiliated organizations, or those of the publisher, the editors and the reviewers. Any product that may be evaluated in this article, or claim that may be made by its manufacturer, is not guaranteed or endorsed by the publisher.

## Abbreviations

Foxp3, transcription factor forkhead box P3; Treg, CD4^+^Foxp3^+^ regulatory T cells; Nrp-1, Neuropilin-1; TGF-β, transforming growth factor-β; IL-10, interleukin-10; IL-2, interleukin-2; iTreg, Treg induced *in vitro* with IL-2 and TGF-β; B6, C57BL/6; IRF4, interferon regulatory factor 4; ALK5i, ALK5 (TGFRI) inhibitor.
