# Supplementary material for: Neuropilin-1 Identifies a New Subpopulation of TGF-β-Induced Foxp3+ Regulatory T Cells With Potent Suppressive Function and Enhanced Stability During Inflammation
Source: Front Immunol. 2022 May 4;13:900139. doi: 10.3389/fimmu.2022.900139 (PMC9114772; doi:10.3389/fimmu.2022.900139)

**Supplemental Figure legends**

**Figure S1.** Nrp-1 was not exclusively expressed in CD4^+^T cells, also higher expressed in NK cells, Macrophage, 11b^+^ cells. Nrp-1 and Helios were detected in different cells from C57BL/6 Foxp3^gfp^ reporter mice. The data indicate the Mean ± SEM of 3 separated experiments.


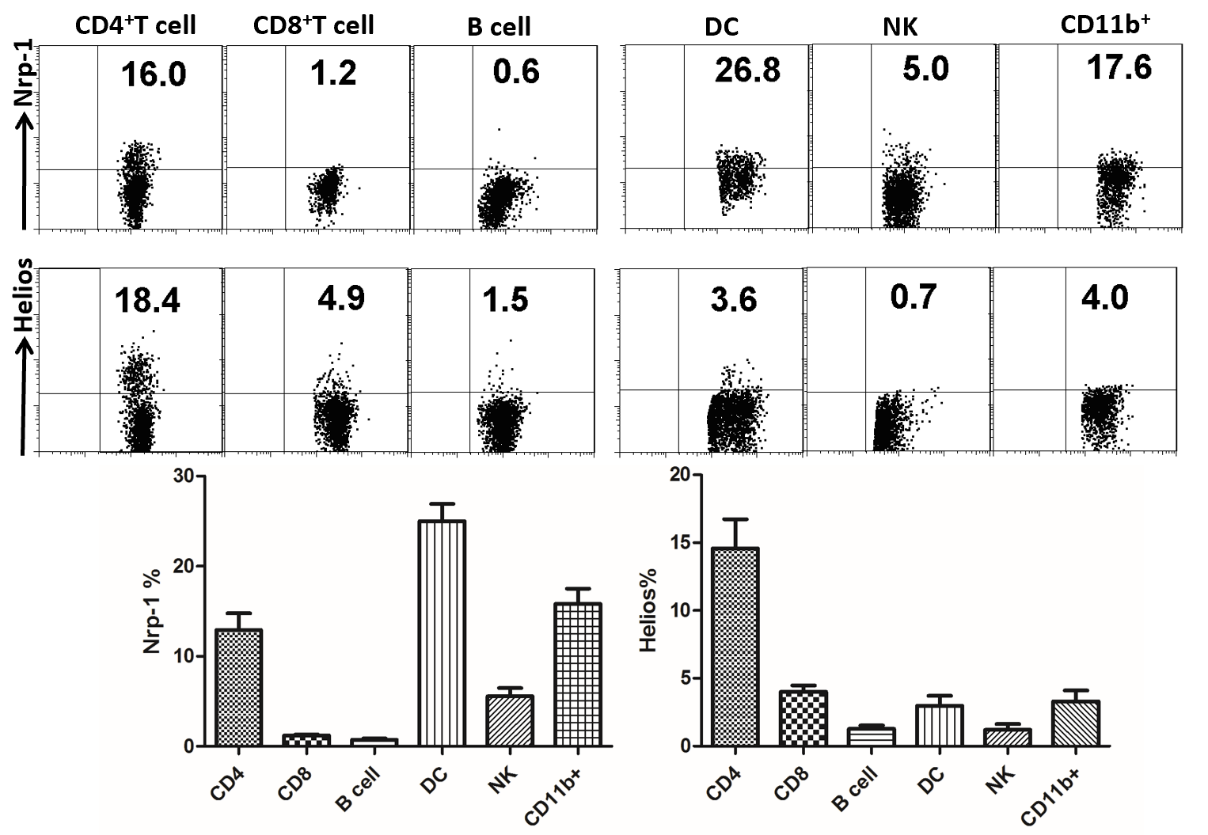


**Figure S2.** Nrp-1 was not an ideal phenotype marker for tTreg. (**A, B and C)** The expression of Nrp-1 and Helios were detected in fresh cells from thymus, lymph node (LN), spleen (SP), blood in B6 Foxp3^gfp^ mice by flow cytometry. The analysis was performed by gating CD4^+^GFP^-^ cells and CD4^+^GFP^+^ cells. Typical FACS plots (up) and summary data (below) were shown. The data indicate the Mean ± SEM of 3 separated experiments (n=6) (**p*<0.05, ***p*<0.01; Nrp-1 and Helios expression in LN, SP, or Blood cells *vs.* Thymus cells).


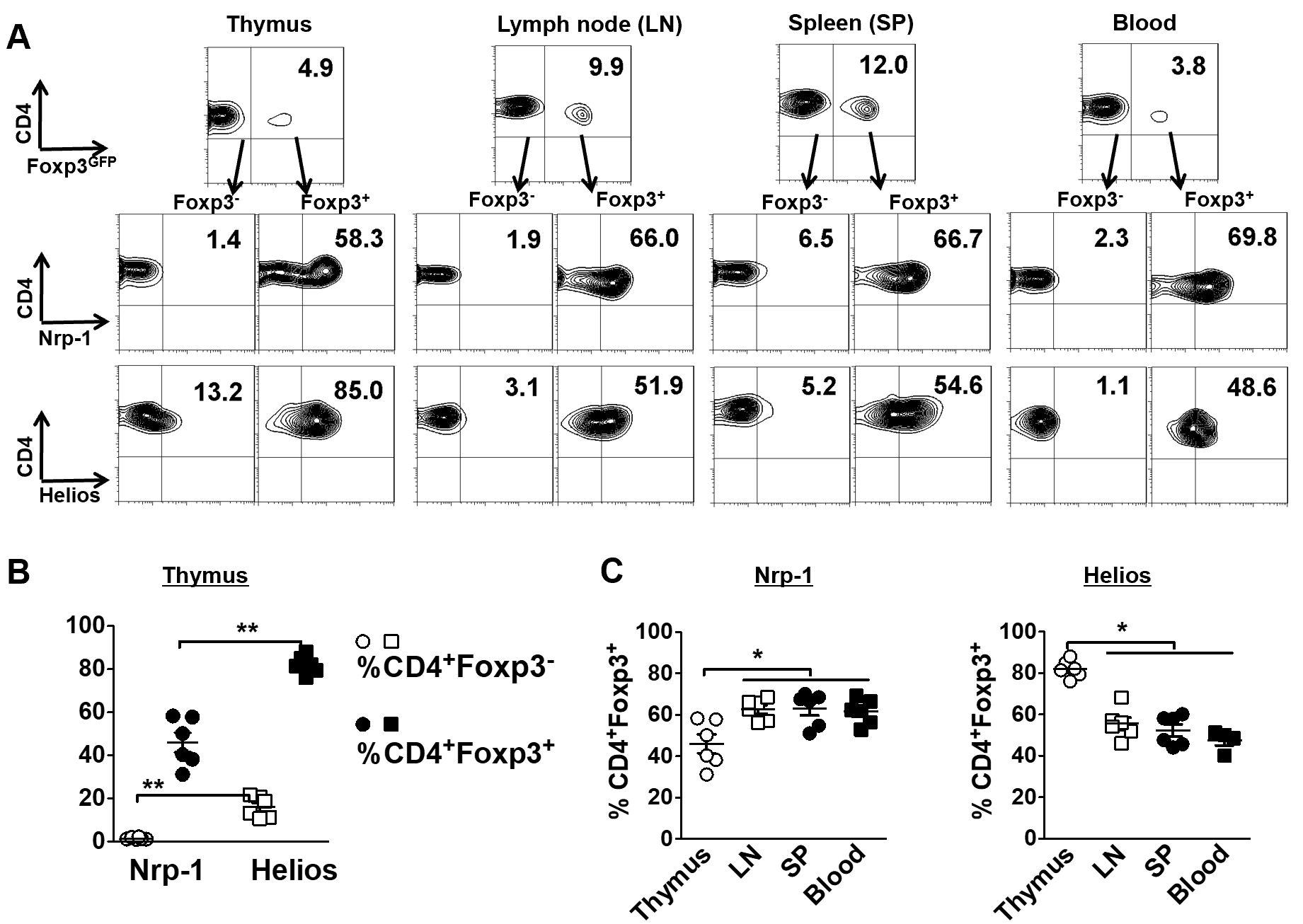


**FIGURE S3.** Nrp-1 was upregulated by TGF-β treatment on CD4^+^iTreg cells *in vitro*, not dependent on smad3 and JNK signal. **(A, B)** iTreg cells were generated from naïve CD4^+^T cells, SIS3 (3μM), JNK inhibitor (10μM), ALK5i (5μM), and DMSO were added to this culture system. The expression of Nrp-1, Helios, and Foxp3-GFP were measured by flow cytometry. Data were presented as the mean ± SEM of three separate experiments. ****p*<0.01 means treatment group *versus* DMSO group.


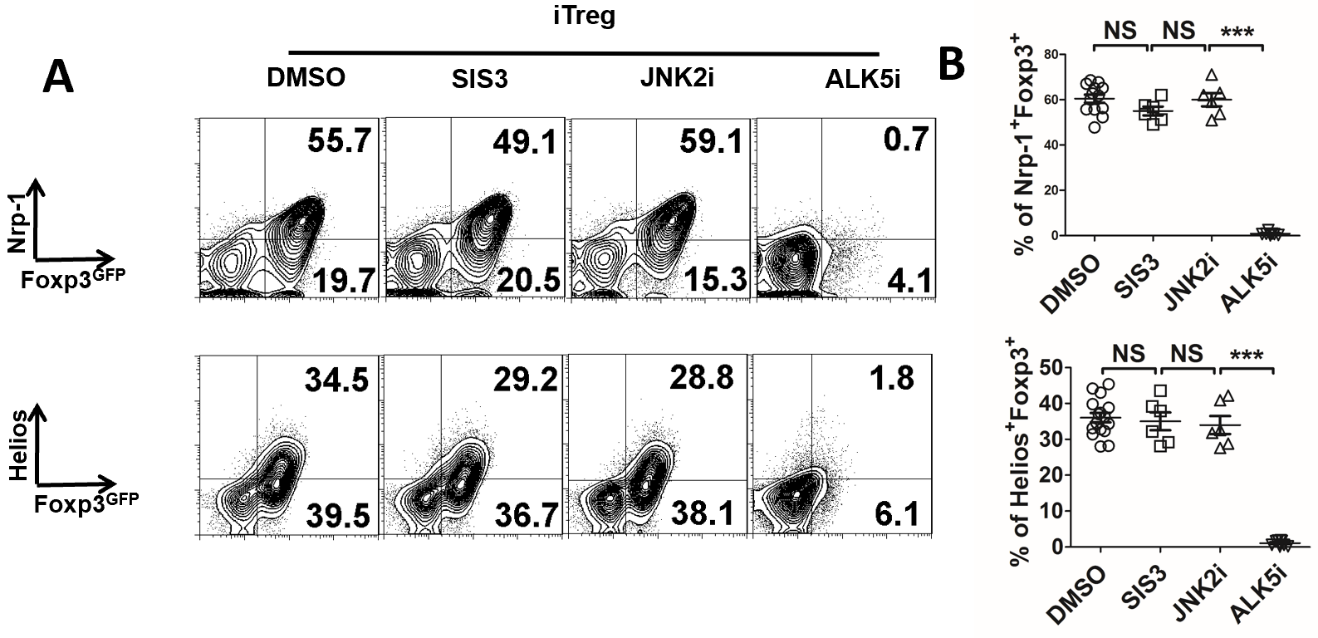


**Figure S4.** Sorting information for Foxp3-GFP^+^Nrp-1^+^iTreg, Foxp3-GFP^+^Nrp-1^-^iTreg, Foxp3-GFP^-^Nrp-1^+^CD4^+^T and Foxp3-GFP^-^Nrp-1^-^CD4^+^T cells.


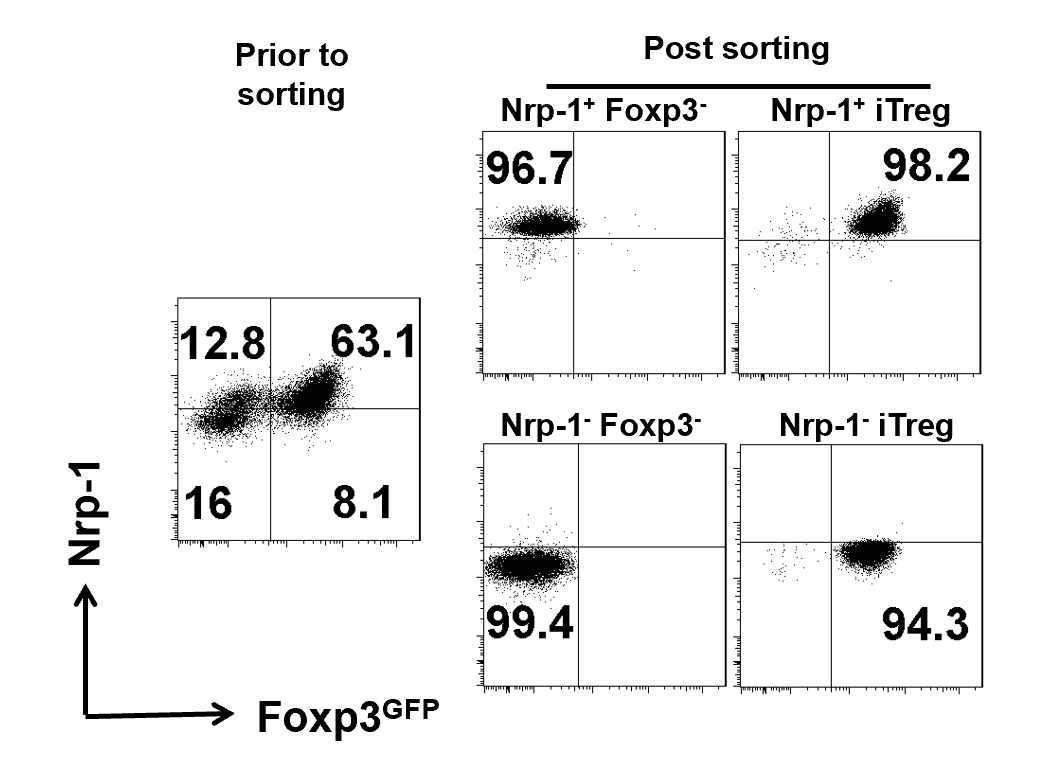

Supplement: Supplementary file 1 [file DataSheet_1.docx]
